# An Analysis of Referrals Done by Primary Care Centers to Tertiary Care Institutions

**DOI:** 10.7759/cureus.62117

**Published:** 2024-06-10

**Authors:** Ramiz Yazıcı, Efe D Bala, Ayşe F Basa Kalafat, Eyüp Sarı, Ishak San, Utku M Kalafat, Rabia B Tapkan, Serkan Doğan

**Affiliations:** 1 Emergency Medicine, İstanbul Kanuni Sultan Suleyman Training and Research Hospital, University of Health Sciences, istanbul, TUR; 2 Emergency Medicine, İstanbul Kanuni Sultan Suleyman Training and Research Hospital, University of Health Sciences, İstanbul, TUR; 3 Pediatrics, Gülhane Faculty of Medicine, University of Health Sciences, Ankara, TUR; 4 Emergency Medicine, Ankara City Hospital, University of Health Sciences, Ankara, TUR

**Keywords:** reasons for referral, healthcare system, primary care, referral patterns, general practice

## Abstract

Background and aim

Primary care is an important element for every healthcare system around the world. Providing and optimizing the connection between the primary care centers and advanced clinical centers is a key concept for a well-functioning healthcare system. Our aim in this study was to analyze and review the referral data of primary care centers located in Ankara, Türkiye.

Materials and methods

We collected the entire referral data from the primary care centers, totaling 8,746 patients between January 1, 2019 and December 31, 2023 by using emergency medical services (EMS) transfer in Ankara. Demographic data, call reasons, transfer centers and transfer-related characteristics of the patients were recorded retrospectively, grouped by year, using EMS data.

Results

Our findings have shown that most of the referrals were made for Turkish citizens with 8,360 (95.6%) (p<0.001). Healthcare centers located in inner city had the most referrals made with 7,087 (81.0%) (p<0.001). Majority of the referrals were made by physicians in family healthcare centers with 6,583 (75.3%) (p<0.001) with chest pain being the most common diagnosis for referral initiation with 1,429 (16.3%) (p<0.001). This was followed by trauma, with 1,172 (13.4%) (p<0.001). Most common cause for trauma was falls with 613 (52.3%) (p<0.001).

Conclusion

Our data revealed important elements of local referral patterns. According to our data, majority of the referrals were made by inner city healthcare facilities. Family healthcare centers formed most of the referral requests. For this reason, strengthening these centers is important to prevent unnecessary resource use and delays.

## Introduction

Creating an accessible and quality health system is the most important part of a sustainable health system. Optimization of health service delivery and establishment of a referral system ensures improvement of medical services and increased service quality through resource management [1]. Optimizing this structural chain of referrals between primary care centers and advanced care centers is important as mishandled referrals can cause delay in specialist care or unnecessary resource allocations for patients who could have been handled at primary care centers with ease [2]. Therefore, referral management should not focus on distributing demand but on making sure that the right patient gets the right amount and type of care at the right time [3].

The Chinese government aimed to spread the medical needs of society throughout the country in a balanced manner by increasing both the number and quality of primary medical institutions. However, it has still been reported that people turn to higher-level institutions rather than primary medical institutions when seeking medical treatment [4]. In Türkiye, according to the data of the Ministry of Health, 340 million applications were made to primary health centers in 2022 [5]. This represents 39.9% of all admissions made to public and private healthcare centers in Türkiye. With such a high number of admissions, it becomes even more important to properly manage the referral process. The aim of hierarchical diagnosis and treatment algorithms published in China in 2015 is to distribute referrals according to the availability of medical institutions for different clinical conditions of patients and to increase the efficiency of the use of advanced clinics [4]. In Türkiye, although everyone can apply to any institution they want, it is aimed to provide health services based on a referral chain.

In order to help optimize the referral process management, we need to understand the referral patterns. In our study, the objective was to analyze the referral data of the capital city, Ankara, collected by the city’s emergency medical services (EMS) between 2019 and 2023.

## Materials and methods

Study design and setting

This study was designed as a retrospective analysis in the city of Ankara. The analysis covers all patients referred from primary care to advanced care centers through EMS between 2019 and 2023. The study was conducted in accordance with the Declaration of Helsinki and approved by the ethics committee permission of the Medical Research Scientific and Ethical Evaluation Board of Ankara Bilkent City Hospital. (Approved no: TABED 1-24-282 and date: 22/05/2024)

This study was conducted between January 1, 2019 and December 31, 2023. A total of 8,746 patients were eligible for this study. Inclusion criteria were being older than 18 years old, being transferred from a primary care center to an advanced care center using public EMS. Exclusion criteria were being under 18 years of age, not being transferred from a primary care center to an advanced care center using public EMS.

Statistical analysis

Data analysis will be done using IBM SPSS 27.0 0 for Windows (Armonk, IBM Corp.) statistical package program. Descriptive statistical methods (frequency, percentage, mean, SD) were used when evaluating study data. The Pearson chi-square test was used to compare qualitative data. The suitability of the data for normal distribution was evaluated with the Kolmogorov-Smirnov test, skewness-kurtosis, and graphical methods (histogram, QQ plot, stem and leaf, boxplot). The independent samples t test and the one-way ANOVA test were used for comparisons of normally distributed quantitative data. In cases where differences were found in multiple comparisons, the post hoc Tukey test and Bonferroni correction were used. The statistical significance level was accepted as p<0.05.

## Results

Of the patients included in the study, 4,311 (49.3%) were women and 4,435 (50.7%) were men, and there was no statistically significant difference (p=0.243). Additionally, there was no significant difference in the average age of female patients by year (p=0.521). However, we have found a statistically significant difference in male gender and the average age of the total participants according to years (p=0.009, p=0.011, respectively). A post hoc Tukey Test was applied to find out which year or years had this difference. The results showed a difference between 2019 and 2022-2023. Additionally, when comparing the data according to sex, we have found a statistically significant difference between men (44.9±25.8) and women (47.1±23.0) in terms of age in the year of 2019 (p=0.048), as we found that the referral age in women is older than their male counterparts. We did not find any significant statistical differences between the genders in other years covered by our study. Our data show that the referral patients were overwhelmingly of Turkish origin. 8,360 (95.6%) of all patients referred were citizens of Türkiye. After evaluating the locations of the primary care centers from which the referrals were made, it was found that the primary care centers within the provincial center had made more referrals to an advanced care center than the primary care centers located in the peripheral towns of the province. We have found that 7,087 (81%) of all referrals between 2019 and 2023 were made by primary care centers in the provincial center. Peripheral primary care centers made only 1,659 (19%) of all referrals between 2019 and 2023 (Table 1).

**Table 1:** Demographic characteristics and transfer characteristics of the groups according to years p<0.05 ^a^: Pearson Chi-squared test was used and data are shown as n (%); ^b^: one-way ANOVA test was used to compare groups according to years (mean ± SD); ^c^: Independent sample t test conducted for the average ages of male and female according to years (mean ± SD)

Characteristics of Participants and Time of Transfers		2019 (n=2,050)	2020 (n=1,456)	2021 (n=1,516)	2022 (n=1,744)	2023 (n=1,980)	Total (n=8,746)	P
Gender	Female	1,002 (48.9)	681 (46.8)	764 (50.4)	875 (50.2)	989 (49.9)	4.311 (49.3)	0.243^ a^
	Male	1,048 (51.1)	775 (53.2)	752 (49.6)	869 (49.8)	991 (50.1)	4.435 (50.7)	
Age (year)		46.0 ± 24.5	46.0 ± 24.5	47.5 ± 24.2	48.8 ± 25.1	47.8 ± 24.0	47.2 ± 24.3	0.009^ b^
Age (year) mean ± SD, Female ^c^		47.1 ± 23.0	47.4 ± 22.9	47.7 ± 23.4	48.9 ± 24.3	47.6 ± 23.3	47.6 ± 23.4	0.521^ b^
Age (year) mean ± SD, Male ^c^		44.9 ± 25.8	47.7 ± 23.8	47.3 ± 24.9	48.6 ± 25.9	48.1 ± 24.7	46.8 ± 25.2	0.011^ b^
P^ c^		0.048	0.842	0.752	0.819	0.639	0.349	--
Country of Origin	Republic of Türkiye	2,011 (98.1)	1,381 (94.8)	1,426 (94.1)	1,647 (94.4)	1,895 (95.7)	8,360 (95.6)	<0.001^ a^
	Others	39 (1.9)	75 (5.2)	90 (5.9)	97 (5.6)	85 (4.3)	386 (4.4)	
District	Central	1,705 (83.2)	1,130 (77.6)	1,229 (81.1)	1,436 (82.3)	1,587 (80.2)	7,087 (81.0)	<0.001^ a^
	Peripheral	345 (16.8)	326 (22.4)	287 (18.9)	308 (17.7)	393 (19.8)	1,659 (19.0)	
Month	January	177 (8.6)	176 (12.1)	84 (5.5)	132 (7.6)	165 (8.3)	734 (8.4)	<0.001^ a^
	February	162 (7.9)	168 (11.5)	98 (6.5)	107 (6.1)	110 (5.6)	645 (7.4)	
	March	189 (9.2)	208 (14.3)	140 (9.2)	155 (8.9)	196 (9.9)	888 (10.2)	
	April	191 (9.3)	132 (9.1)	110 (7.3)	125 (7.2)	154 (7.8)	712 (8.1)	
	May	162 (7.9)	75 (5.2)	80 (5.3)	136 (7.8)	176 (8.9)	629 (7.2)	
	June	136 (6.6)	150 (10.3)	177 (11.7)	143 (8.2)	132 (6.7)	738 (8.4)	
	July	159 (7.8)	128 (8.8)	150 (9.9)	103 (5.9)	178 (9.0)	718 (8.2)	
	August	149 (7.3)	83 (5.7)	132 (8.7)	152 (8.7)	174 (8.8)	690 (7.9)	
	September	173 (8.4)	59 (4.1)	110 (7.3)	148 (8.5)	175 (8.8)	665 (7.6)	
	October	179 (8.7)	94 (6.5)	132 (8.7)	168 (9.6)	178 (9.0)	751 (8.6)	
	November	158 (7.7)	73 (5.0)	152 (10.0)	179 (10.3)	169 (8.5)	731 (8.4)	
	December	215 (10.5)	110 (7.6)	151 (10.0)	196 (11.2)	173 (8.7)	845 (9.7)	

The evaluation of another set of data reveals that most of the referrals were made by family healthcare centers with 6,583 (75.3%) of all referrals, followed by pharmacies and apothecaries with 1,189 (13.6%) of all referrals. Integrated healthcare centers, refugee healthcare services, community healthcare centers, workplace physicians, private clinics, and other centers together only made up 974 (11.1%) of all referrals. Medical reasons such as syncope, chest pain, or palpitations were the most common cause of referrals with 7,752 (88.6%). This was followed by various accidents 676 (7.7%), soft tissue traumas 136 (1.6%), traffic accidents 135 (1.5%), workplace accidents 28 (0.3%) and suicides 19 (0.2%) (Table 2).

**Table 2:** Referral centers and their reasons by year p<0.05

Referral Centers and Reasons		2019 (n=2,050)	2020 (n=1,456)	2021 (n=1,516)	2022 (n=1,744)	2023 (n=1,980)	Total (n=8,746)	P
Type of Primary Care Centers	Family Healthcare Centers	1,656 (80.8)	1,126 (77.3)	1,120 (73.9)	1,257 (72.1)	1,424 (71.9)	6,583 (75.3)	<0.001
	Pharmacies	281 (13.7)	160 (11.0)	211 (13.9)	239 (13.7)	298 (15.1)	1,189 (13.6)	
	Integrated Healthcare Centers	26 (1.3)	38 (2.6)	50 (3.3)	56 (3.2)	109 (5.5)	279 (3.2)	
	Refugee Healthcare Centers	17 (0.8)	37 (2.5)	36 (2.4)	54 (3.1)	35 (1.8)	179 (2.0)	
	Community Healthcare Centers	18 (0.9)	35 (2.4)	60 (4.0)	22 (1.3)	35 (1.8)	170 (1.9)	
	Workplace Physicians	34 (1.7)	28 (1.9)	15 (1.0)	20 (1.1)	39 (2.0)	136 (1.6)	
	Private Clinics	7 (0.3)	5 (0.3)	6 (0.4)	51 (2.9)	22 (1.1)	91 (1.0)	
	Home Health Care	1 (0.0)	18 (1.2)	15 (1.0)	35 (2.0)	6 (0.3)	75 (0.9)	
	District Healthcare Directiories	9 (0.4)	8 (0.5)	3 (0.2)	8 (0.5)	9 (0.5)	37 (0.4)	
	Occupational Safety and Health Centers	1 (0.0)	1 (0.1)	0 (0.0)	2 (0.1)	3 (0.2)	7 (0.1)	
Reasons for Referrals to Tertiary Care Institutions	Medical	1,791 (87.4)	1,299 (89.2)	1,348 (88.9)	1,563 (89.6)	1,751 (88.4)	7,752 (88.6)	0.003
	Other Accidents	185 (9.0)	111 (7.6)	115 (7.6)	131 (7.5)	134 (6.8)	676 (7.7)	
	Soft Tissue Trauma	45 (2.2)	16 (1.1)	23 (1.5)	20 (1.1)	32 (1.6)	136 (1.6)	
	Traffic Accidents	22 (1.1)	20 (1.4)	25 (1.6)	22 (1.3)	46 (2.3)	135 (1.5)	
	Work Accidents	5 (0.2)	6 (0.4)	3 (0.2)	2 (0.1)	12 (0.6)	28 (0.3)	
	Suicides	2 (0.1)	4 (0.3)	2 (0.1)	6 (0.3)	5 (0.3)	19 (0.2)	

Another set of data shows that most referrals were made to training and research hospitals as tertiary care centers in Türkiye, with 4,257 (53.7%) of all referrals being made to these centers. The training and research hospitals were followed by secondary care centers and state hospitals in this regard with 2,772 (35%) of all referrals. 563 (7.1%) of all referrals were made to university hospitals, and lastly, private clinic referrals were much rarer, accounting for about 335 (4.2%) of all referrals (Table 3). These same data also show that 687 (7.9%) of all referral requests were rejected for various reasons. 99 (1.1%) of all referral requests were terminated after the EMS squad on site resolved the situation themselves and declared that there was no need for a referral to an advanced healthcare center. 27 (0.3%) of the cases were transferred to their homes after a referral request was made. Six (0.1%) of all cases were declared exitus on site (Table 3). As mentioned above, medical conditions were the most common cause of a referral request. To go deeper into this subject, our database also collected the preliminary diagnosis of these patients. We detected that 2,330 (26.6%) of all referrals were made with various preliminary diagnosis, followed by chest pain with 1,429 (16.3%), trauma with 1,172 (13.4%), hypertension with 1,059 (12.1%) and dyspnea with 470 (5.4%) of all cases, respectively.

**Table 3:** Call results and initial diagnoses by year p<0.05

Call Results and Initial Diagnosis		2019 (n=2,050)	2020 (n=1,456)	2021 (n=1,516)	2022 (n=1,744)	2023 (n=1,980)	Total (n=8,746)	P
Result	Transfer–Hospitals	1,920 (93.7)	1,350 (92.7)	1,340 (88.4)	1,544 (88.5)	1,773 (89.5)	7,927 (90.6)	<0.001
	Training and Research Hospitals	1,048 (54.6)	750 (55.6)	715 (53.4)	774 (50.1)	970 (54.7)	4.257 (53.7)	
	State Hospitals	626 (32.6)	472 (35.0)	493 (36.8)	589 (38.1)	592 (33.4)	2.772 (35.0)	
	Universities	170 (8.9)	84 (6.2)	87 (6.5)	100 (6.5)	122 (6.9)	563 (7.1)	
	Private Clinic	76 (4.0)	44 (3.3)	45 (3.4)	81 (5.2)	89 (5.0)	335 (4.2)	
	Transfer Rejection	113 (5.5)	92 (6.3)	148 (9.8)	144 (8.3)	190 (9.6)	687 (7.9)	
	Intervention on Site	17 (0.8)	9 (0.6)	20 (1.3)	41 (2.4)	12 (0.6)	99 (1.1)	
	Transfer to Home	0 (0.0)	4 (0.3)	6 (0.4)	13 (0.7)	4 (0.2)	27 (0.3)	
	Exitus on Site	0 (0.0)	1 (0.1)	2 (0.1)	2 (0.1)	1 (0.1)	6 (0.1)	
Complaints of Patients	Chest Pain	340 (16.6)	218 (15.0)	243 (16.0)	297 (17.0)	331 (16.7)	1.429 (16.3)	<0.001
	Trauma	305 (14.9)	185 (12.7)	193 (12.7)	213 (12.2)	276 (13.9)	1,172 (13.4)	
	Hypertension	272 (13.3)	140 (9.6)	191 (12.6)	233 (13.4)	223 (11.3)	1,059 (12.1)	
	Dyspnea	88 (4.3)	102 (7.0)	73 (4.8)	104 (6.0)	103 (5.2)	470 (5.4)	
	Abdominal Pain	80 (3.9)	51 (3.5)	44 (2.9)	77 (4.4)	91 (4.6)	343 (3.9)	
	Nausea and Vomiting	78 (3.8)	29 (2.0)	56 (3.7)	81 (4.6)	93 (4.7)	337 (3.9)	
	Syncope and Fainting	87 (4.2)	39 (2.7)	55 (3.6)	53 (3.0)	75 (3.8)	309 (3.5)	
	Dizziness and Vertigo	71 (3.5)	33 (2.3)	41 (2.7)	56 (3.2)	67 (3.4)	268 (3.1)	
	Medical observation for suspected diseases and conditions	0 (0.0)	205 (14.1)	67 (4.4)	42 (2.4)	2 (0.1)	316 (3.6)	
	Anaphlaxis	39 (1.9)	24 (1.6)	36 (2.4)	41 (2.4)	51 (2.6)	191 (2.2)	
	Fever	48 (2.3)	26 (1.8)	19 (1.3)	23 (1.3)	34 (1.7)	150 (1.7)	
	Cardiac Arrhytmia	37 (1.8)	33 (2.3)	24 (1.6)	34 (1.9)	24 (1.2)	152 (1.7)	
	Hyperglicemia	20 (1.0)	16 (1.1)	21 (1.4)	18 (1.0)	28 (1.4)	103 (1.2)	
	Pregnancy and Labor	17 (0.8)	18 (1.2)	12 (0.8)	13 (0.7)	16 (0.8)	76 (0.9)	
	Self-Harm	5 (0.2)	8 (0.5)	3 (0.2)	10 (0.6)	6 (0.3)	32 (0.4)	
	Chronic Kidney Failiure	0 (0.0)	3 (0.2)	4 (0.3)	2 (0.1)	0 (0.0)	9 (0.1)	
	Others	563 (27.5)	326 (22.4)	434 (28.6)	447 (25.6)	560 (28.3)	2,330 (26.6)	

Our data on trauma patients, which made up approximately 1,172 (13.4%) of all referral requests between 2018 and 2023, reveal that the most frequent reason for referral cases trauma was falls with 613 (52.3%). This was followed by soft tissue traumas with 287 (24.5%), traffic accidents with 133 (11.3%), assault with 77 (6.6%), animal bites with 57 (4.9%) and amputations with five (0.4%) of all cases, respectively. (Table 4)

**Table 4:** Subanalysis of trauma diagnoses by years p<0.05

Trauma Diagnosis	2019 (n=2,050)	2020 (n=1,456)	2021 (n=1,516)	2022 (n=1,744)	2023 (n=1,980)	Total (n=8,746)	P
Trauma	305 (14.9)	185 (12.7)	193 (12.7)	213 (12.2)	276 (13.9)	1.172 (13.4)	<0.001
Fall	151 (49.5)	96 (51.9)	99 (51.3)	127 (59.6)	140 (50.7)	613 (52.3)	0.055
Soft Tissue Trauma	78 (25.6)	45 (24.3)	48 (24.9)	51 (23.9)	65 (23.6)	287 (24.5)	
Traffic Accidents	23 (7.5)	24 (13.0)	27 (14.0)	18 (8.5)	41 (14.9)	133 (11.3)	
Assault	32 (10.5)	10 (5.4)	10 (5.2)	9 (4.2)	16 (5.8)	77 (6.6)	
Animal Bite	20 (6.6)	10 (5.4)	8 (4.1)	8 (3.8)	11 (4.0)	57 (4.9)	
Amputation	1 (0.3)	0 (0.0)	1 (0.5)	0 (0.0)	3 (1.1)	5 (0.4)	

## Discussion

In our study, our aim was to evaluate the statistical data of referrals made to primary care centers, collected by the EMS of the capital city of Türkiye, Ankara, between 2019 and 2023.

We found that the overwhelming majority of the referrals were made for Turkish citizens with 8,360 (95.6%). According to the Directorate of Immigration, 89,343 documented refugees were living in Ankara, accounting for 1.52% of the population [6]. We have also found that referrals from refugee healthcare centers have made up 179 (2.0%) of all referrals in Ankara. In a study conducted by Chang, the health disparities of immigrants in the United States were discussed. It was stated that immigrants have consistently been affected by negative determinants of health such as marginalization, poverty, stigmatization, communication problems, poverty and house and food insecurity [7]. Our data has found that when compared to the total population ratio, more referrals were made for non-Turkish citizens than Turkish citizens. While the existence of refugee healthcare centers and services for immigrants is an encouraging factor for immigrants to seek medical help in times of need, high referral rates can also be a sign for problems in communication, as stated by the study conducted by Chang [7].

We have found that most referral requests were made from primary care centers located in the province center and not from towns in the periphery of the province. Similarly, it has been reported that living in the city and having more access to a specialist positively affects referral rates [8]. Another review conducted by Cancino has reported that inner cities or provincial centers, as we have mentioned, have a more high-risk patient profile, due to dense population, competing demands, poor distribution of primary care providers, and financial limitations [9].

Our data showed that most of the referrals were made by family healthcare centers with 6,583 (75.3%) of all referrals being made by family healthcare physicians. The physician rosters of these centers often consist of general practitioners rather than trained family physicians. Therefore, results varying by two-three times in family physician referral rates have been reported [10]. A study conducted by Jantsch et al. in 2022 has shown that residency training in Family Medicine reduces referral rates drastically [11]. A meta-analysis by Elrashidi et al. has reported that the colocation of specialists in primary care settings can also provide more efficient care and prevent fragmentation of care [12]. Apart from the training of family physicians, differences in the patient population for which they are responsible and the rate of public admission to primary health centers may cause variability in referral rates.

Our study revealed the most common specific reason for referral as chest pain with 1,429 (16.3%) of all referrals made as a result of chest pain. A study conducted by Blinkenberg et al. in 2022 also reported chest pain as one of the most frequent reasons for referral, forming 14,077 (5%) of all referrals [13]. However, their study has detected the most common referral cause as abdominal pain with 21.260 (8%). Our data also report abdominal pain as one of the most common referral causes with 343 (3.9%). The disparity of between these results could be caused by the lower threshold of primary care physicians to refer patients with chest pain. In a study conducted Buch et al, it was stated that only 75 (7.8%) of all patients with chest pain were referred to cardiology departments in United States [14]. While excessive referrals to cardiology departments are found to be unnecessary, this could be the reason behind the dramatic difference between the unusually high rates of referral of patients with chest pain in our study group.

Another common reason for referral was trauma. We analyzed the reasons for trauma and found that the most common reason for referred trauma patients was falls. A similar study conducted by Luggya et al. in 2022 reports traffic accidents as the most common cause for referral to advanced care centers [15]. These results seem to differ between different studies, as regional dynamics around the world heavily influence the mechanism of the most common type of injury admitted to the advanced care centers.

In 2019, Covid-19 hit healthcare systems around the world. With an increased influx of patients and strained resources, primary care providers helped immensely to manage this crisis and responded to the problems created by this pandemic, as stated by DeVoe et al. [16]. The data from our study showed that even when the pandemic was at its peak, referrals from primary care centers did not overwhelm the capacity of advanced care centers, thus showing the great work done by primary care physicians, similarly to those mentioned in the article above.

Our data have shown that conditions and symptoms such as chest pain or abdominal pain were among the most frequently referred conditions. The potential diagnosis of these patients could lead to a situation in which a specialist could be needed in an emergency setting. As stated by Reynolds et al., the global demand for surgical and anesthesia services exceeds the supply, therefore, a well-organized and effective healthcare system should have strong links between primary care providers and emergency services, especially to serve those in need of care outside of office hours [17]. The results of our data analysis support this approach as we can see conditions that can result in a need for advanced care specialists are referred considerably more than other conditions.

This study provides information to health system designers about referrals from primary care centers. This information will contribute to the necessary updates and optimization for the sustainability of the system. Additionally, examining the reasons for referral needed by family physicians will be beneficial in their training.

As this was a retrospective cross-sectional observational study, we did not have access to the patients at the time of their evaluation. We analyzed the data after the process was completed. This helped us eliminate bias by blinding the primary physician who evaluated the patient, but it also limited our ability to get full details about the patient. We tried to minimize bias by having different sets of physicians and researchers to collect data and analyze the results.

## Conclusions

The results of our study support the maintenance of stronger ties between primary health care services and advanced health centers. Ensuring standardization in the education of family physicians and complying with certain rules for referred patients will make significant contributions to the functioning of the health system.
